# Analyzing the impact of bicycle geometry and cargo loading on the rideability and safety of cargo bikes: An investigative study

**DOI:** 10.1016/j.heliyon.2024.e29524

**Published:** 2024-04-11

**Authors:** Milan Paudel, Fook Fah Yap

**Affiliations:** a Transport Research Centre @ NTU (TRC@NTU), Nanyang Technological University, Singapore, 50 Nanyang Avenue, 639798; b School of Mechanical and Aerospace Engineering, Nanyang Technological University, Singapore, 50 Nanyang Avenue, 639798

**Keywords:** Cargo bikes, Cargo load, Safety, Rideability, Long-tail bike, Long-john bike

## Abstract

**Introduction:**

Electric cargo bikes have become popular for transporting goods and people due to their small size and strong carrying capacity. However, the way they perform, handle, and operate safely can be affected by the weight of the cargo, where it is placed on the bike, and the bike's design.

**Method:**

This paper analyzes the rideability and safety of eight different cargo bikes representing three different design categories, Retrofitted, Long-john, and Long-tail bikes, also considering three different cargo loading locations. We quantitatively examined the rideability by computing the minimum speed for self-stability, the maximum possible acceleration and deceleration without losing wheel-ground contact, the handlebar torque for steady-state turning, and the force required to overcome obstacles. The effect of using powerful motorized wheels has also been discussed.

**Results:**

Long-john cargo bikes are unstable for lightweight cargo loads, more sensitive to cargo loads, and therefore may not be suitable for riding in narrow, crowded spaces like footpaths. Moreover, retrofitted cargo bikes should only be used to carry lightweight cargo as a combination of heavy cargo load and a powerful rear wheel motor poses a potential risk of accidents. Long-tail cargo bikes are less affected by changes to the cargo load and are thus safer than retrofitted bikes. Their relatively compact length also makes for a smaller turning radius.

**Conclusion:**

Rideability and safe handling of the cargo bikes strongly depend on the bike design and load and loading position. Retrofitted bikes are not suitable for carrying heavy loads and any load at the front has an adverse effect on the overall rideability and safety.

**Practical application:**

The results highlight the benefits and limitations of different cargo bike designs and, therefore, could have implications for the cargo bike manufacturers, service providers, and policymakers.

## 1 Introduction

A cargo bike is a specially modified bicycle for transporting heavy goods, a task that is challenging for standard or conventional bikes [1]. The fundamental structure of cargo bikes shares similarities with standard bicycles, featuring at least two wheels, a main frame with a saddle, a steering mechanism comprising a handlebar, and, additionally, cargo bikes are equipped with cargo carriers or racks to securely transport goods or loads [2]. The compact size and heavier load-bearing capacity to vehicle weight ratio have made cargo bikes appealing to many freight companies or last-mile logistics providers [1,2]. Advancements in technology, particularly in battery-powered assistance systems and compact, efficient drive systems, have propelled cargo bikes into powerful vehicles. Wheels with hub motors or mid-drive e-cargo bikes help users cover a longer distance and easily overcome challenging terrains.

The growing concern about environmental issues such as climate change, pollution, and traffic congestion has contributed to the rising popularity of cargo bikes [1,3]. Governments and policymakers are trying to promote green logistics through the adoption of zero-emission vehicles [1]. Cargo bikes have been put forward as an alternative to traditional motor vehicles for urban delivery in many countries [3]. Food delivery companies, grocery stores, and short-distance freight companies are increasingly embracing cargo bikes for transporting goods and services [4]. These bikes require less space for loading and storage and are more maneuverable in crowded and congested areas compared to motor vehicles. Some cargo bikes also have seats for parents and caretakers to transport children [5,6], making them versatile and able to meet a variety of needs. As a result, there are many different designs of cargo bikes available on the market.

**Fig. 1:**
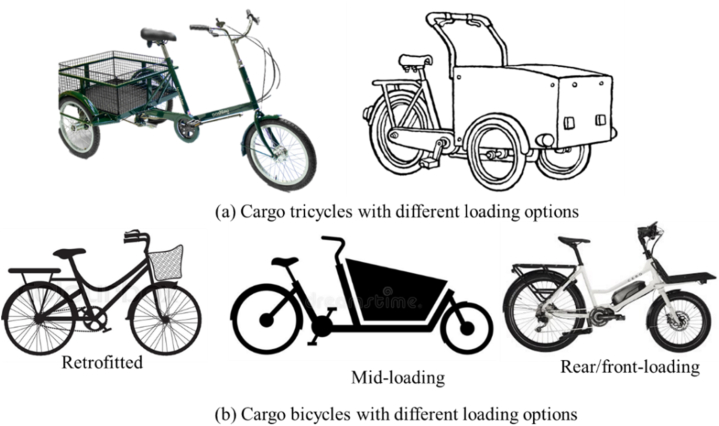
Some typical designs of cargo bikes with different cargo loading locations (a) cargo tricycles (b) cargo bicycles.

Some typical designs of cargo bikes based on wheel arrangements and cargo loading options are shown in Fig. 1. There are typically three loading positions: front-loading, rear-loading, and mid-loading. Furthermore, tricycles have two different wheel arrangements: Delta (one wheel at the front) and Tadpole (two wheels at the front).

With the increasing popularity of cargo bikes, perceived safety emerges a potential barrier to achieving the goal of green logistics [7]. Cargo bikes, with or without load, should be easy to control and safe for riders so that they can concentrate more on traffic and surrounding environments [8]. However, cargo bikes are much heavier, longer, and wider than conventional bicycles. Consequently, cargo bikes are not as agile as conventional bikes [9]. Moreover, cargo loads affect the weight distribution, impacting the dynamic behavior of the bike. Heavy cargo loads could significantly affect the stability, handling, braking and setting-off performance [9]. Moreover, cargo bikes with longer wheelbases could be challenging to negotiate turns in narrow shared paths [4,10].

Although considered a sustainable option for urban delivery [11], with an array of cargo bike designs and growing demand, there are only a handful of reports and news [12,13], and few published articles focusing on the design and safety aspects of electric bicycles or e-cargo bikes used by couriers or messengers [14]. Recent studies on cargo bikes primarily explore travel patterns, environmental and health benefits, impacts and advantages on logistics, as well as riders' perceptions and behavioral aspects [11,15,16]. These studies largely rely on user surveys. Some research indicates a positive correlation between risky behaviors and attitudes among riders, inadequate infrastructure, and the risk of accidents [17,18]. Surveys in China have identified traffic violations such as running red lights, improper lane changes, and mobile phone use while cycling as contributing factors to accident risks [19,20]. In Italy, the presence of partial crossings and bicycle signs has been linked to intersection-related accidents [17]. Other factors, including high speeds and rapid accelerations of bikes, have also been associated with crashes [17,18]. Furthermore, research has delved into psychological aspects such as risk acceptance, perception, and workload [21], along with demographic factors like gender and age, in relation to the safe operation of cargo bikes [6,17]. Notably, safety concerns have been shown to affect adoption and ridership, especially among women [6].

Some studies have also investigated injuries, collision and accidents risks involving e-bikes and cargo e-bikes. Dennerlein and Meeker [14] conducted research with 113 courier bicycle participants, of whom 70 % of the courier bike riders reported suffering from injuries leading to off days at work, and 55 % had to visit hospitals or clinics seeking treatment. A study in China reported that e-bike crashes increased yearly, reaching 56,200 collisions, 8400 deaths, and 63,500 injuries from 2013 to 2017 [18]. Another research from Norway, highlighted the effect of weather condition on maneuverability and effort required to ride the cargo-bike [3]. Similar research by Malik et al. also suggested a strong interaction effect between weather and travel distance [15]. Insufficient braking and possible safety risks have also been reported reported by D'hondt et al. [10]. Moreover, some attention have also been given to the safety of the cargo bike with retrofitted child seats [13].

However, there is no holistic scientific research on the design of the cargo bike and how the different loading can affect the dynamic performance and safety of such bikes. With the increasing popularity of both motorized and nonmotorized cargo bikes, it is essential to understand their safety performance.

This paper presents the performance evaluation of different designs and analyzes the influence of cargo loading on the rideability of cargo bikes. Here, rideability is defined as the ability of the rider to stably ride the bike and perform different maneuvers with ease [22]. The paper focuses on cargo bicycles (two inline wheel configurations) as they are laterally unstable compared to tricycles. Tricycles have unique wheel arrangements that enable them to remain upright even when stationary. Unlike tricycles, bicycles are single-track vehicles with two inline wheels that require certain forward speed to remain upright [23,24]. In other words, these single-track vehicles are dynamically unstable at low speed, and once the bike surpasses a threshold speed known as “weave critical speed”, the bike becomes self-stable [23]. A bike can maintain a straight upright motion by itself and does not require any external control to maintain balance in a self-stable state.

**Fig. 2:**
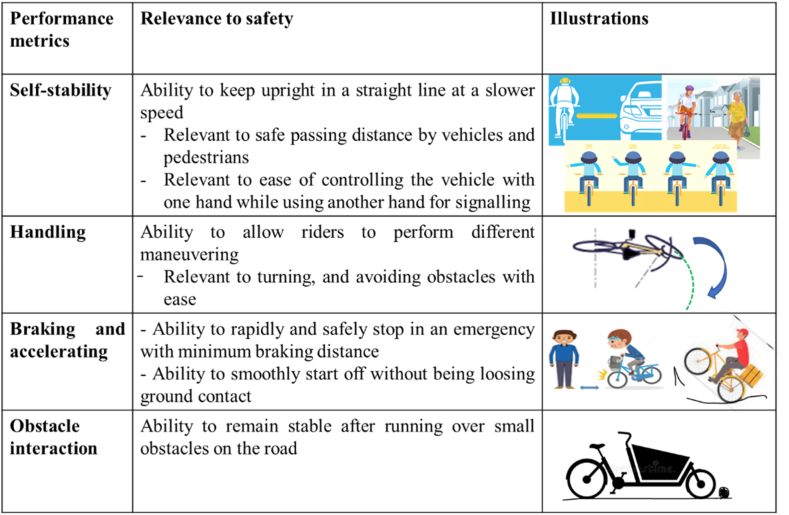
An illustration of safety metrics and their safety implications.

In addition to the self-stability, handling, braking deceleration, setting-off acceleration, and obstacle interaction are some key metrics that contribute to the safety of cargo bikes. Fig. 2 illustrates the safety relevance of the abovementioned performance metrics.

This paper is arranged into the following six sections. The second section briefly describes the basic design features and parameters of different cargo bikes. The third section presents the theoretical background related to performance metrics. The fourth section presents the results, and the fifth section discusses the results and tries to relate the results to safety performance. Finally, the conclusion is presented in the sixth section.

## 2 Basic design specification of cargo bikes

This section presents the specifications of various cargo bike designs. In recent years, the growth of last-mile logistics services has resulted in a surge of riders using retrofitted cargo carriers on their bicycles or e-bikes for transporting goods. These carriers can be affixed to the front or rear of the vehicle, enabling riders to transport a diverse range of goods. Such retrofitted cargo bicycles have also been considered in this analysis.

**Fig. 3:**
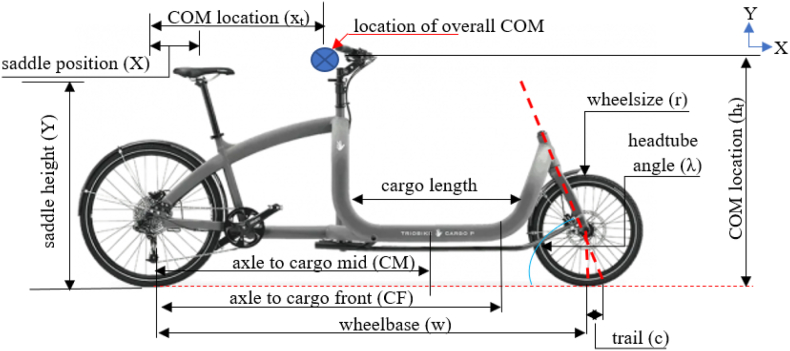
Basic geometrical parameters of a cargo bike.

**Fig. 4:**
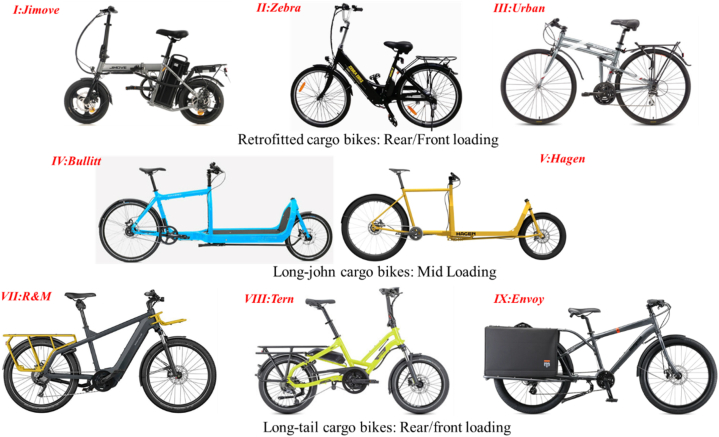
Eight selected models of cargo bikes considered for analysis.

The geometrical and structural specifications of the cargo bikes were analyzed to comprehend their design features. Fig. 3 illustrates the basic geometric parameters of a cargo bike. A total of 8 cargo bikes, representing three different categories, namely (i) Retrofitted cargo bikes, (ii) Long-john mid-loading cargo bikes, and (iii) Long-tail rear/front-loading long-tail cargo bikes, have been considered in this analysis. Eight selected models representing three different categories of bikes are shown in Fig. 4.

A simple method, as adopted by Paudel and Yap [25], has been used to obtain the complete geometrical parameters of cargo bikes. The design specifications, such as wheelbase, overall length, and wheel size, were extracted from the official website, and the undistorted images of the bike were imported into the CAD software to determine the remaining geometrical parameters. Table 1 provides a compilation of geometrical parameters for each selected cargo bike.

**Table 1:** Design parameters of eight cargo bikes selected for analysis.

Cargo bike	Retrofitted			Mid loading		Rear/front loading		
	I	II	III	IV	V	VII	VIII	IX
Model	Jimove	Zebra	Urban	Bullitt	Hagen	R&M^a^	Tern	Envoy
Weight, kg	20	19.5	13.2	23.5	26.5	28.5	22.8	18.5
Wheelbase, m	0.89	1.037	1.04	1.85	1.81	1.2	1.165	1.28
Front wheel size, inch	14	24	29	20	20	26	20	26
Rear wheel size, inch	14	24	29	26	29	26	20	26
Headtube angle, deg	71.5	71	71	70	69.23	69.5	71	70
Trail, mm	41.5	72.5	67.48	58	74.5	86	50	70
Saddle position: X, m	0.203	0.21	0.21	0.201	0.235	0.34	0.36	0.42
Saddle height: Y, m	0.719	0.90	0.95	0.95	1.01	0.98	0.95	0.93
Axle to cargo front, m	0.057	0.10	0.12	1.54	1.5	0.285	0.275	0.3
Axle to cargo mid, m	−0.055	−0.056	0.047	1.2	1.19	0	0	−0.06
Drive^b^	RWD	RWD	HP	HP	HP	MD	MD	HP

a R&M: Raise and Muller Multicharger cargo bike.

b Drive: RWD: Rear wheel drive, HP: Human-powered MD: Mid drive.

The following observations are made about the design of cargo bikes from selected models in Table 1.

1. Retrofitted cargo bikes exhibit the shortest wheelbase (range: 0.89–1.037 m), while the Long-john cargo bikes have the longest wheelbase (range: 1.81–2.06 m). Long-tail cargo bikes have a wheelbase slightly longer than Retrofitted bikes.
2. Long-john and Retrofitted cargo bikes have similar saddle positions (X) from the rear wheel-ground contact point. Conversely, Long-tail cargo bikes have saddle positions situated further ahead of the rear wheel-ground contact point.
3. Human-powered Long-john cargo bikes weigh heavier than their Retrofitted and Long-tail counterparts (Mid drive bikes vs. Urban vs. Envoy). Nonetheless, battery-assisted bikes are much heavier than manual drives (Urban vs. Tern).
4. Long-john cargo bikes typically incorporate larger wheels (≥24 inches) at the rear and smaller wheels (≤20-inches) at the front, possibly to achieve a longer cargo space.
5. The saddle height for all the bikes is similar except for the smallest 14-inch wheel E-bike (I: Jimove) which features a lower height (0.72 m) compared to other bike models (0.9–1 m).
6. Retrofitted and Long-tail cargo bikes have cargo locations closer to the rear wheel axle. Long-john bikes have cargo locations more than a meter ahead of the rear wheel axle (axle to cargo mid).

In addition to the aforementioned observations, Long-john cargo bikes are designed to carry heavier loads exceeding 150 kg [26,27], while, Long-tail cargo bikes typically accommodate 50 kg at the rear rack and 10–20 kg at the front basket [28]. The cargo capacity of Retrofitted bikes is generally less defined but could range from 20 to 50 kg at the rear and 5–10 kg at the front. Furthermore, the weight of the cargo bikes also depends on battery capacity and the accessories attached to the bike. In this analysis, we have used the baseline model of the bikes with minimal accessories attached to them. The design specifications and observations from Table 1 will be used to analyze the safety performance of cargo bikes.

## 3 Theoretical background

This section presents the theoretical background related to performance metrics based on which the safety performance will be analyzed.

### 3.1 Self-stability

Self-stability of single-track vehicles has intrigued many researchers for centuries [[29], [30], [31]]. In 2007, Meijaard et al. [23] published the benchmark equations of motion based on rigid body dynamics and validated the equations with experiments. The bicycle is modeled using a combination of four rigid bodies: front wheel, rear wheel, front steering frame (handlebar + fork), and rear frame. A rigid rider can be attached to the rear frame maintaining the riding posture [32]. The benchmark equations of motion for bicycles can be written as equation (1). It represents two coupled second-order differential equations defined for lean angle *(φ*) and steering angle *(δ),* and constant velocity*(v).* The coefficients *M, C*_*1,*_ *K*_*0,*_ and *K*_*2*_ represent the mass, damping, and stiffness matrices defined in terms of 25 design variables which can be divided into two parts: (i) geometrical parameters and (ii) mass-related parameters. The equation of motion can be solved for eigenvalues to identify the minimum speed for which the vehicle is self-stable [33].

(1) $$M\left(\begin{array}{c} \ddot{\emptyset }\\ \ddot{\delta } \end{array}\right)+vC_{1}\left(\begin{array}{c} \dot{\emptyset }\\ \dot{\delta } \end{array}\right)+\left(gK_{0}+v^{2}K_{2}\right)\left(\begin{array}{c} \emptyset \\ \delta \end{array}\right)=\left[\begin{array}{c} T_{\emptyset }\\ T_{\delta } \end{array}\right]$$

### 3.2 Braking and acceleration

Riders frequently need to accelerate and decelerate while operating the bikes. Acceleration shifts the weight toward the rear wheel, and deceleration shifts the weight toward the front wheel. Excessive braking or acceleration can cause a wheel to lose traction and potentially lead to an accident. The maximum acceleration and deceleration a bike can achieve without losing wheel-ground contact are given by equations (2), (3)) [34].

(2) $$Maximum\;braking\;deceleration\;without\;losing\;rear\;wheel-ground\;contact=\frac{g\left(w-x_{T}\right)}{h_{T}}$$

(3) $$Maximum\;acceleration\;without\;losing\;front\;wheel-ground\;contact=\frac{g\left(x_{T}\right)}{h_{T}}$$

Where, $g,w,x_{T},and\;h_{T}$ represent the acceleration due to gravity, wheelbase, and the horizontal and vertical position of the center of mass (COM) of the cargo bike system (bike + rider + cargo load) measured from rear wheel-ground contact, respectively.

If the cargo bike is equipped with a motorized wheel at the rear axle that can provide a torque of τ Nm, Equation (3) is modified as:

(4) $$Maximum\;acceleration\;without\;losing\;front\;wheel-ground\;contact=\frac{g\left(x_{T}\right)}{h_{T}}-\frac{\;\tau }{m_{t}h_{t}}$$

Where $m_{t}$ represents the total mass of the cargo bike system.

### 3.3 Handling

Although handling is associated with a strong subjective perception, it is often characterized by the vehicle's response to a rider's control actions [35]. A bike with good handling performance helps a rider to efficiently execute different maneuvers. Schwab et al. [36] has also suggested that the dynamics of an uncontrolled bicycle is closely related to its handling performance.

Steady-state turning is one of the most common handling tests [12]. In steady-state turning, both lean and steering rates are zero. Equation (1) can be modified to represent a steady state turning [37].

(5) $$Lean\;Equation:g*K_{0\emptyset \emptyset }\emptyset +{(v}^{2}*K_{2\emptyset \delta }+g*K_{0\emptyset \delta })\delta =0$$

(6) $$Steer\;Equation:g*K_{0\emptyset \delta }\emptyset +{(v}^{2}*K_{2\delta \delta }+g*K_{0\delta \delta })\delta =T_{\delta }$$

Furthermore, the steering angle could be replaced with a turning radius as:

(7) turningradius(Rc)=w/cosλ*δ

Where, $K_{xx}$ $T_{\delta }$, and λ represent elements of the stiffness matrices in equation (1), handlebar torque, and headtube angle, respectively.

### 3.4 Obstacle interaction

Any obstacle, such as a pothole, road hump, or step, can potentially destabilize a bike. Furthermore, whenever a wheel encounters an obstacle and starts to roll over it, the rider may experience a jarring shock through the handlebar, which has adverse impact the overall riding experience. How a bike interacts with obstacles is crucial for safety, as a significant number of single-vehicle bike accidents are caused by road obstacles [38,39]. Equation (8) represents a simple formula to calculate the horizontal force required to overcome an obstacle of a certain height.

(8) $$\text{Horizontal}\;\text{force}\;\text{to}\;\text{overcome}\;\text{obstacle}\;\left(F\right)=\frac{m_{T}*g*x_{t}*\cos\;\alpha }{\sin\;\alpha \left(w+x\right)-\cos\;\alpha \left(r-h\right)}$$

Furthermore,

(9) $$\cos\;\alpha =\frac{\sqrt{h\left(2r-h\right)}}{r}$$

(10) $$\sin\;\alpha =\frac{r-h}{r}$$

(11) $$x=\sqrt{h\left(2r-h\right)}$$

Where *r* and *h* represent the front wheel radius and obstacle height, respectively.

## 4 Results

This section examines the effect of cargo weights and their placements on the safety performance of various cargo bikes. We considered a maximum of 50 kg cargo load at the rear and mid-rack and a maximum of 10 kg cargo load at the front basket. Before adding cargo loads, we modeled all of the bikes in SolidWorks [40] maintaining their geometrical shapes and tube thickness were selected carefully to accurately reflect the frame weight. The bike frames' center of mass (COM) and moment of inertia (MOI) information were extracted from SolidWorks.

The cargo was modeled as a rigid rectangular box, with dimensions varying based on the carried load. The moment of inertia properties of the cargo loads were estimated using a standard formula for the cuboid. The rear and mid cargo loads are merged with the bike's rear frame. We also considered two different loading options for the front cargo load: (i) the front basket attached to the handlebar and (ii) the front basket attached to the headtube. For the first cargo loading option, the mass and MOI properties of cargo are merged with the front steering frame (handlebar + fork), which rotates as we steer the handlebar. Conversely, the mass-related parameters, for the second loading option, were merged with the bike's rear frame.

**Fig. 5:**
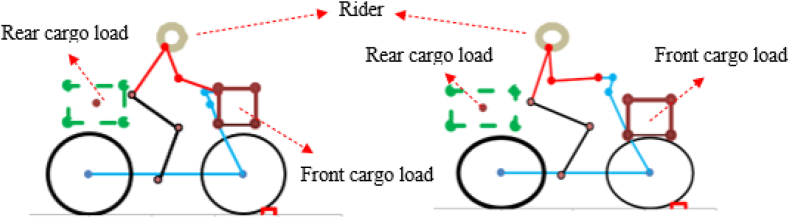
Schematic representation of cargo bike, cargo load, and rider's posture.

A rigid rider, weighing 72 kg and measuring 1.83 m in height, was attached to the rear frame, maintaining the riding posture. Fig. 5 shows the schematic diagram of the bicycle, the rider's posture, and cargo loads. It should be noted that no front load has been added either to the handlebar or the headtube of Long-john cargo bikes while analyzing the performance. This is because the Long-john cargo bikes lack the option for front-loading.

**Fig. 6:**
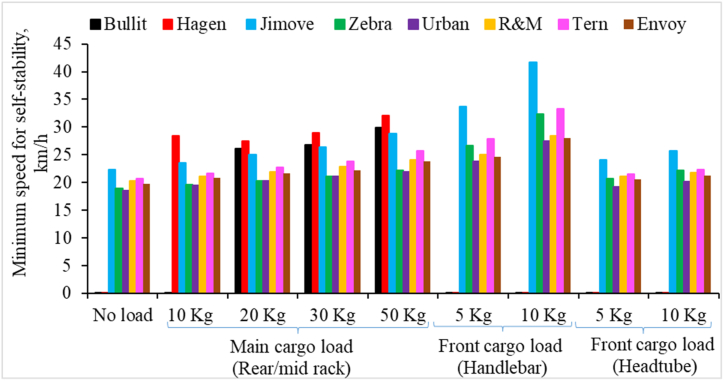
Minimum speed for self-stability for cargo bikes with increasing cargo loads at different loading locations.

Fig. 6 compares the minimum speed required for self-stability for different cargo bikes. The following observations are made from Fig. 6.

1. Mid-loading Long-john cargo bikes are unstable when no load is added to the bike. The Bullitt bike showed self-stability when a 20 kg cargo load was added to it. Similarly, The Hagen cargo bike showed self-stability when adding a 10 kg cargo load. Notably, both Long-john bikes require a higher minimum speed for self-stability compared to Retrofitted and Long-tail bikes.
2. The self-stability has shifted towards a higher speed as we increased the cargo load, irrespective of the loading locations.
3. A significant increase in minimum speed for self-stability is observed when the cargo load is added to the front basket attached to the handlebar.
4. Retrofitted cargo bikes with bigger wheels (Zebra and Urban) have a lower minimum speed for self-stability than Long-tail cargo bikes. However, the smaller wheel retrofitted bike (Jimove) has shown a higher minimum speed for self-stability.

**Fig. 7:**
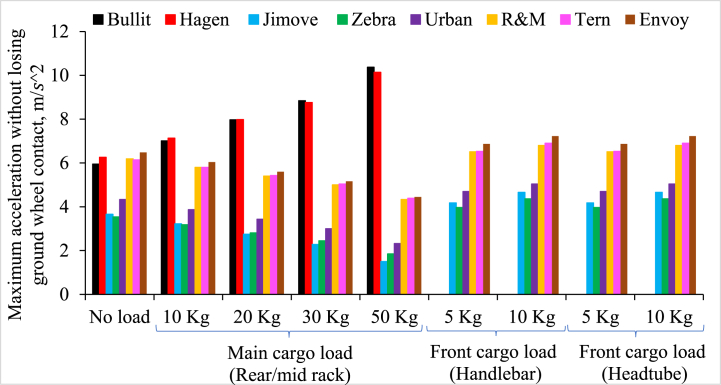
Maximum acceleration for different cargo bikes without losing front wheel-ground contact and influence of increasing cargo load at different cargo loading locations.

Fig. 7 illustrates the maximum acceleration that these cargo bikes can achieve without losing front wheel-ground contact. If the acceleration values surpass the limit presented in Fig. 7, the bike's front wheel will lose contact with the ground and potentially leading to an accident. The following observations are made from Fig. 7.

1. Long-john and Long-tail cargo models exhibit significantly values for maximum acceleration compared Retrofitted cargo bikes, both with or without cargo loads.
2. Maximum acceleration values reduce as we increase the cargo load at the rear rack of Retrofitted and Long-tail cargo bikes.
3. Conversely, the maximum acceleration values rise with an increase in the cargo load for mid-loading Long-john bikes.

**Fig. 8:**
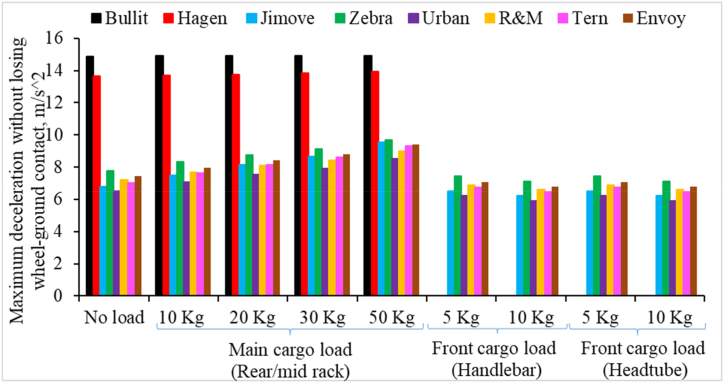
Maximum deceleration for different cargo bikes without losing rear wheel-ground contact and influence of increasing cargo load at different cargo loading locations.

Fig. 8 shows the maximum deceleration that the different models of cargo bikes can achieve without losing the rear wheel-ground contact. The following observations are made from Fig. 8.

1. Long-tail and Retrofitted cargo models have similar values for maximum deceleration. In comparison, Long-john cargo bikes have a significantly higher threshold for maximum deceleration.
2. Maximum deceleration values increase when cargo load is added to Long-tail and Retrofitted cargo bikes.
3. Surprisingly, maximum deceleration values for Long-john cargo bikes do not change significantly with increasing cargo load.
4. A cargo load to the front (handlebar/headtube) slightly reduced the maximum deceleration values.

**Fig. 9:**
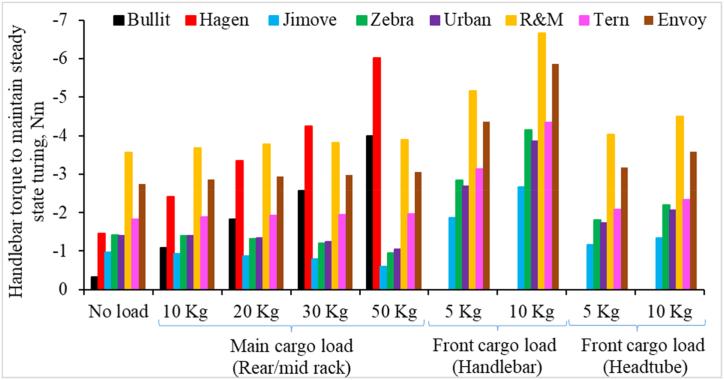
Handlebar torque required by cargo bikes for steady-state turning and influence of increasing cargo load at different loading locations.

Again, Fig. 9 shows the handlebar torque by the rider to maintain a steady state turning of 3 m radius at 20 km/h speed. The following observations were made from Fig. 9.

1. All cargo bikes need negative handlebar torque for steady-state turning. Here, the negative torque represents the torque in the opposite direction of the turn. For e.g., if the rider is trying a counter-clockwise turn, the rider has to apply clockwise torque to prevent oversteering and maintain the turn.
2. Handlebar torque increases sharply in magnitude and becomes more negative when the cargo is loaded onto the front basket attached to the handlebar. Even a small front cargo load on the handlebar basket significantly amplifies the handlebar torque. However, the cargo load on the front basket attached to the headtube does not have a similar adverse effect.
3. With no cargo loads, Long-tail cargo bikes need more negative handlebar torque than Retrofitted and Long-john cargo models. Surprisingly, the handlebar torque values remain almost the same even when a heavy cargo load is added to the rear rack of a Long-tail cargo bike.
4. The handlebar torque values reduces in magnitude and becomes less negative, when cargo is loaded onto the rear rack of Retrofitted cargo bikes.
5. The handlebar torque value increases in magnitude and becomes more negative when the load is added to the mid-rack of the Long-john cargo bike. Even a small increase in the cargo load on Long-john cargo bikes causes a significant increase in handlebar torque.

**Fig. 10:**
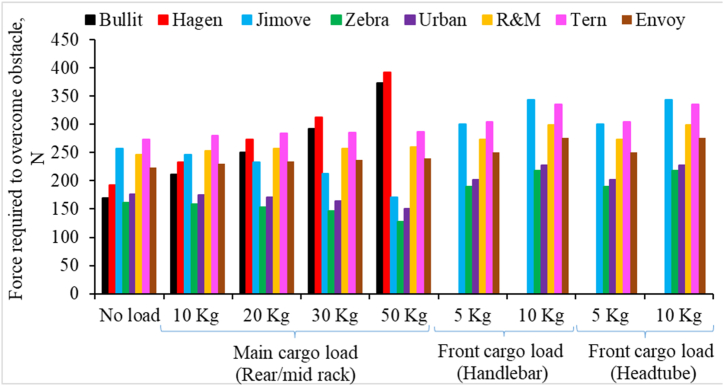
Horizontal force required for different cargo bikes to overcome an obstacle of 4 cm height and influence of increasing cargo load at different loading locations.

Finally, Fig. 10 shows the horizontal force required to overcome an obstacle of 4 cm height. The obstacle represents a small stationary object, steps, or potholes of 4 cm in height. The following observations are made from Fig. 10.

1. Small-wheeled Retrofitted cargo bikes (I: Jimove) demand a substantially higher force to overcome an obstacle compared to other Retrofitted counterparts with bigger wheels (Zebra and Urban).
2. For Long-john cargo bikes, the force required to overcome obstacles increases more rapidly with increasing cargo load compared to other categories of cargo bikes.
3. The force necessary to overcome obstacles is slightly reduced for Retrofitted cargo bikes when the cargo load is increased. An opposite effect was observed for Long-john and Long-tail cargo bikes.
4. Even a small load at the front significantly increases an effort to overcome obstacles. The effect of front loading remains consistent regardless of whether the front load is added to the handlebar or the headtube basket.

**Fig. 11:**
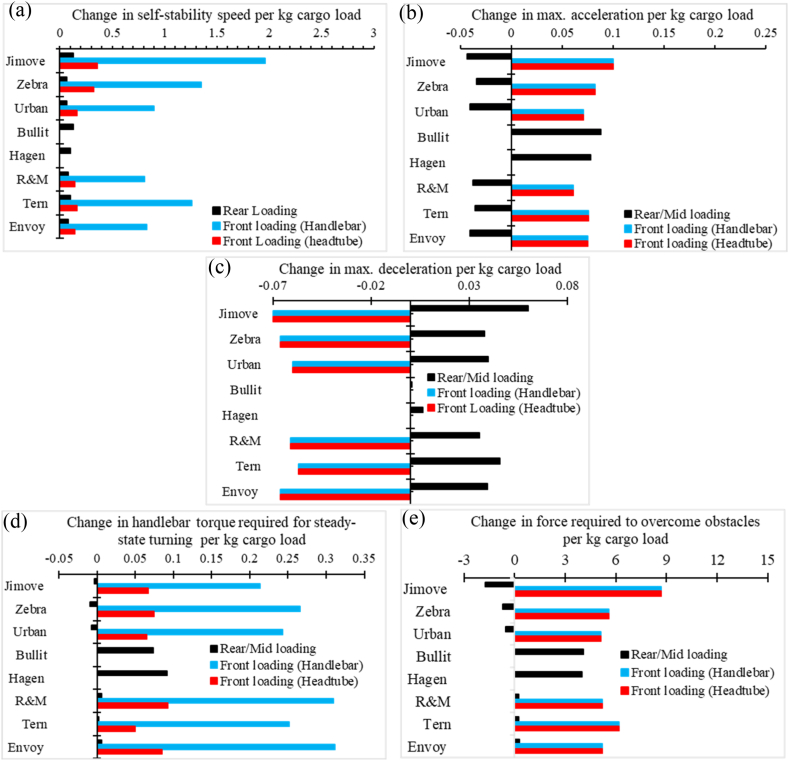
Rate of change on performance metrics per kg cargo load at different cargo loading locations.

We further analyzed our results to understand how sensitive the different models of cargo bikes are to the cargo loads and loading locations. We calculated increment rates of performance metrics for each cargo bike per kg change in cargo load. Results are presented in Fig. 11.

Here, positive increment rates suggest that the performance metrics will increase when the cargo load is increased at that particular loading location. A higher increment rate shows that the cargo bike model is more sensitive to the cargo load and cargo location. The following observations are made from Fig. 11.

1. Performance metrics such as self-stability, maximum acceleration and deceleration, handlebar torque, and force required to overcome obstacles are more sensitive to front-cargo loading than rear-cargo loading.
2. When comparing the two front-loading options, performance metrics are more sensitive to the cargo loads on the handlebar basket than on the headtube basket. Also, the effect of cargo loading on the handlebar basket has been found to be more adverse than the cargo load on the headtube basket.
3. Performance metrics such as acceleration, handlebar torque, and force required to overcome obstacles are more sensitive to the cargo loads for Long-john bikes than other categories of cargo bikes.
4. The maximum deceleration for Long-john cargo bikes is surprisingly less sensitive to added cargo loads.
5. Retrofitted cargo bikes are more sensitive to cargo loading than Long-tail cargo bikes, although both are rear-loading cargo bikes.

## 5 Discussion

Retrofitted, Long-john, and Long-tail models differ in saddle and cargo loading location, which result in different weight distribution and moment of inertia of the overall cargo bike system (bike + rider + cargo load). Moreover, the geometrical parameters such as wheel radius, wheelbase, steering angle, and trail length (Fig. 3) also influence the dynamic performance of the cargo bikes.

### 5.1 Self-stability performance

The results have shown that the unique design of the Long-john cargo bikes is not self-stable when lightweight cargo loads (0–10 kg) are added. A similar result has been reported by Williams [41]. However, heavier cargo loads (>10 kg) may change the weight distribution, and Long-tail bikes may show self-stability.

Cargo bikes with a smaller front wheel and a longer steering trail length require higher speed to be self-stable. Similar observations have been reported on typical bicycle designs with smaller wheels and longer trail lengths [25,32]. At low speed, such cargo bikes require greater control input from the rider in order to maintain balance. An unstable bike could be perceived as twitchy and nervous, and any disturbance from an obstacle or uneven terrain at the front could easily be amplified [32] elevating the risk of accidents.

### 5.2 Acceleration and deceleration performance

The maximum allowable acceleration and deceleration are a function of the weight distribution of the cargo bike [34]. A center of mass (COM) location towards the rear wheel axle improves the braking performance but reduces the aceleration performance and vice-versa. Similarly, a higher COM location is detrimental to both acceleration and braking performance. Cargo loads influence the acceleration and deceleration performance as it tends to shift the COM location. Cargo load at the front shifts the COM towards the front of the bike, improving the acceleration performance but decreasing the maximum allowable deceleration.

The COM of Long-john bikes, with no cargo load, is positioned around the middle of the bike length and close to the ground, resulting in superior acceleration and deceleration performance compared to Retrofitted and Long-tail cargo bikes. Adding a cargo load to the mid rack of the Long-john bikes enhances the maximum allowable acceleration values. This is because the cargo load added to the mid rack of the long-john bikes further lowers the COM and shifts its COM of the overall system towards the front end, increasing the distance 'xt' in Equation (3). However, the maximum deceleration values remain relatively similar, regardless of the added cargo load, as depicted in Fig. 8, Fig. 11. This is because the maximum deceleration performance depends on the ratio between the horizontal distance of the COM measured from the front wheel contact point (${w-x}_{t})$ and the vertical distance of the COM. For Long-John cargo bikes, the horizontal location (${w-x}_{t})$ and the vertical location of the COM decrease at a similar rate when a cargo load is added.

In contrast, the maximum allowable acceleration decreases with added cargo load for Retrofitted and Long-tail cargo bikes as the COM of the cargo bike system shifts towards the rear wheel axle. This effect is opposite to that of a Long-john cargo bike as explained above. Furthermore, Retrofitted cargo bikes have much lower value for maximum acceleration compared to Long-tail cargo bikes. This is because Long-tail cargo bikes are designed such that the rider position is shifted further forward from the rear wheel axle to maximize the cargo carrying capacity. This shifts the overall COM location further away from the rear wheel axle. The COM location, coupled with a slightly longer wheelbase of Long-tail cargo bikes enables them to achieve higher maximum acceleration than Retrofitted cargo bikes.

It is also worth noting that Retrofitted cargo carriers are much shorter than the cargo carriers on Long-tail cargo bikes. Therefore, large cargo goods on Retrofitted cargo carriers are likely to be placed behind the rear wheel axle. Any cargo load behind the rear wheel axle will exert a moment that reduces the front wheel-ground contact reaction force. Therefore, if a large and heavy cargo load is placed on Retrofitted cargo bikes, there is a greater risk of the front wheel losing ground contact.

### 5.3 Turning and obstacle overcoming performance

For steady state turning, the handlebar torque depends on the front wheel size and mass parameters, overall weight distribution, head tube angle, and trail length [37]. All the cargo bikes have a negative handlebar torque. Previous studies have indicated that a negative handlebar torque is desirable on single-track vehicles, as it aids in maintaining a circular path and keeping the bike upright in case of sudden handlebar control loss [34].

Our analysis suggests that Long-john cargo bikes require less handlebar torque to perform steady-state turning maneuvers when no cargo load is added. However, the handlebar torque increases sharply in magnitude and becomes more negative when a cargo load is added. Moreover, they require a greater turning radius due to their longer wheelbase. This greatly compromises the agility of the Long-john cargo bike. This characteristic may make Long-john cargo bikes challenging to maneuver on very narrow footpaths or crowded shared paths.

On the other hand, Retrofitted cargo bikes with shorter wheelbases offer greater agility for navigating narrow and crowded paths. Furthermore, Long-tail cargo bikes are found to require more handlebar torque compared to retrofitted cargo bikes. The results could be attributed to the bigger, wider, and heavier front wheels, bike weight, forward positioning of the rider, and longer trail distance. However, by carefully tuning the front steering design, especially the head tube angle and trail length, the handlebar torque for the Long-tail cargo bikes could be improved [25].

Moreover, our study recommends avoiding the placement of cargo loads on the front basket attached to the handlebar, as it significantly increases (i) the speed required for self-stability, (ii) handlebar torque, and (iii) force needed to overcome an obstacle.

### 5.4 Effect of motorized wheel

**Fig. 12:**
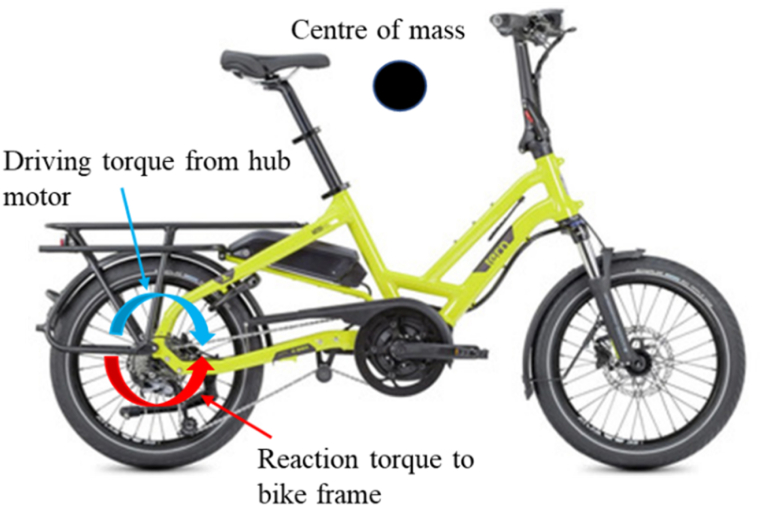
Illustration of reaction torque from motorized wheel on bike frame.

There has been a recent trend for using battery-powered pedal-assist systems on bikes. This innovation enables riders to travel longer distances and tackle steep terrain with less exertion. Smaller wheels, compact and portable designs of battery-powered bikes with provision to retrofit cargo carriers (I: Jimove, Zebra bike) are becoming very popular among platform-based delivery riders, especially in Asia [42]. One of the major concerns with battery-powered rear hub motorized wheel bikes is that it reduces the maximum allowable acceleration of the bikes as it exerts a reaction moment on the frame, as shown in Fig. 12.

Most hub motors on e-bikes could generate a torque ranging from 50 to 60 Nm, with some powerful hub motors capable of generating torque around 80–120 Nm [43]. Considering an average reaction torque of 60 Nm on the bike frame from the hub motor and using Equation (4), the maximum possible acceleration values for cargo bikes are calculated. Table 2 presents the maximum acceleration when Retrofitted and Long-tail cargo bikes are equipped with a motorized wheel at the rear axle.

**Table 2:** Maximum acceleration values for rear-loading cargo bikes due to motorized wheel.

Cargo bikes	Maximum acceleration values (m/s^2^)			
	Without motor	With battery-powered motor (60Nm)		
	Cargo load: 0 kg	Cargo load: 0 kg	Cargo load: 20 kg	Cargo load: 50 kg
Jimove	3.66	2.70	2.04	1.05
Zebra	3.54	2.80	2.19	1.36
Urban	4.33	3.57	2.82	1.84
R&M	6.19	5.60	7.41	3.88
Tern	6.14	5.46	7.35	3.89
Envoy	6.48	5.40	4.80	3.97

Heavy cargo on the rear rack and reaction torque from the hub motor significantly reduce the maximum allowable acceleration for Retrofitted cargo bikes (Jimove, Zebra, and Urban). Typically, 500–1000 W motorized wheels can accelerate to between 1.3 and 1.42 m/s^2^ for a total laden bike weight of 121–150 kg [44], similar to having a 50 kg cargo load in our analysis. The reported experimental acceleration range is higher than the maximum acceleration values for Retrofitted bikes with a rear motorized wheel and 50 kg cargo load at the rear rack; refer to Table 2 (Jimove, Zebra). Considering the experimental acceleration and the design features, Retrofitted cargo bikes are recommended to carry a maximum rear load of not more than 15 kg.

A combination of a more powerful motorized rear wheel on Retrofitted cargo bikes, a lightweight rider, and cargo weight could easily surpass the maximum allowable acceleration. In addition, some of the retrofitted bikes have a very short wheelbase, of between 750 and 800 mm in length, and the rider position is further towards the rear wheel axle. If the starting acceleration is higher, such retrofitted cargo bikes may suffer from a sudden loss of wheel-ground contact which may lead to a loss of control. Therefore, retrofitted cargo bikes should not be used with heavier cargo loads and powerful motorized wheels. Furthermore, a high acceleration on single-track vehicles has been found to reduce the self-stability range [22]. In the worst case, the self-stability could disappear due to high acceleration. Therefore, e-cargo bikes should be carefully tuned to provide gradual power assistance to the rider without affecting the rideability and safety of the riders and the surrounding. Further studies are required to identify the maximum permissible power rating and the optimum acceleration strategies for different power assist levels, especially during setting-off.

Table 3 summarizes the general safety performance of cargo bikes. Long-john bikes lacks self-stability without cargo load and exhibit poor low-speed self-stability. Moreover, small-wheeled Retrofitted bikes do not have good low-speed self-stability compared to big-wheel retrofitted cargo bikes. Retrofitted and Long-tail cargo bikes with bigger wheels have overall better low-speed self-stability than Long-john bikes. On the other hand, Long-tail and Long-john cargo bikes have better acceleration and deceleration performance than Retrofitted cargo bikes. Moreover, a shorter wheelbase makes retrofitted bikes more agile and easy to perform quick turning.

**Table 3:** Summary of safety indices and recommended cargo weight limit.

	Retrofitted	Long-tail	Long-john
General performance			
Low speed stability	Medium	Good	Poor
Hard braking	Poor	Medium	Good
Acceleration	Poor	Medium	Good
Turning/Manoeuvring	Good	Medium	Poor
**Cargo weight recommendation**			
Mid/Read loading	0–15 kg	15–50 kg	>50 kg
Front loading at headtube	Not recommended	Up to 5 kg	Not applicable
Front loading at handlebar	Not recommended	Not recommended	Not applicable

Table 3 also outlines the cargo weight recommendations based on the analysis. Considering the factors such as the use of a rear hub motor, short wheelbase, small wheels, and structural integrity of the cargo rack, Retrofitted cargo bikes are recommended to carry a load of not more than 15 kg on the rear rack. As the Retrofitted cargo bikes with small wheels are susceptible to cargo load at the front compared to other categories of cargo bikes, front cargo load is not recommended on Retrofitted cargo bikes. On the other hand, Long-tail and Long-john bikes are designed for a heavier load. However, for a cargo load between 20 kg and 50 kg, Long-tail cargo bikes are recommended as they have better stability and handling and require less force to overcome compared to Long-john cargo bikes. Furthermore, the cargo-bikes should also consider the fact that the kinetic energy of the overall system (cargo bike, rider, and load) also increases with the speed and the cargo load on it. That means the cargo bikes must produce a higher braking torque to stop the cargo bike with the shortest possible braking distance. Therefore, cargo bikes that are designed to carry heavy loads (Long tail and Long John cargo bikes) could be equipped with disc brakes with larger rotors to achieve higher braking torque. Furthermore, the ABS braking system could be used to further enhance braking safety [38].

In addition, e-cargo bikes with mid-drive motors are also available on the market and are believed to provide a natural feel of riding a bike compared to the sensation of being either pulled or pushed, as is the case with hub-motor drives. Mid-drive e-bikes are considered to have better performance, as the rider can change the speed and torque using the drivetrains, especially when going uphill. Moreover, the mid-drive also helps to evenly distribute the bike's load compared to hub motors. Another difference between mid-drive motor and rear hub motor is the speed control. The hub motor driven e-bikes operate at some fixed speed corresponding to the assist level. Therefore, they do not allow riders to properly control the speed. Whereas, mid-drive cargo bikes provide assist on the torque provided by riders, therefore, the speed of the bike depends on the cadence of the rider. In other words, mid-drive cargo bikes allow riders to control the speed which could be considered safer. However, all these features come at a higher cost compared to e-bikes with hub motors.

## 6 Conclusion

This paper explores the impact of frame designs and additional cargo loads on the dynamic performance of various cargo bikes. A total of eight cargo bikes representing different categories of cargo bikes: (i) Retrofitted, (ii) Long-john, and (iii) Long-tail, have been considered. Furthermore, three different cargo loading locations: (i) rear-rack, (ii) mid-rack, and (iii) front basket (handlebar attached and headtube attached), have also been considered. The dynamic performance of the selected cargo bikes is assessed based on self-stability, maximum acceleration and deceleration, handlebar torque for steady-state turning, and ability to overcome obstacles.

Results have shown that increasing the cargo load adversely shifts the self-stability towards a higher speed, regardless of the cargo loading locations. The acceleration performance of Long-john cargo bikes improves with increasing cargo loads; however, an opposite effect was seen for Retrofitted and Long-tail cargo bikes. Surprisingly, the maximum permissible deceleration for Long-john cargo bikes remains relatively unaffected by increasing cargo loads. Moreover, even a lightweight cargo load at the front significantly affects the performance metrics. Our results have recommended avoiding front cargo loading, especially on the carrier affixed to the handlebars, as it has an adverse effect on the overall rideability by increasing the speed for self-stability, the handlebar torque for turning, and the force required to overcome obstacles.

Our analysis has found that Retrofitted cargo bikes are not designed to carry a heavy load and should not be equipped with a powerful motorized rear wheel. On the other hand, they are more suitable for use as lightweight cargo carriers on narrow, shared paths because their short wheelbase, compactness and lightweight make them more agile than the other types of cargo bike.

The unique design of Long-john cargo bikes with a much longer wheelbase is unstable when no cargo load is added. Long-john cargo bikes are designed to carry heavy loads and are less agile as they require a larger turning radius. The minimum speed for self-stability, the required handlebar torque, and the force to overcome obstacles increases sharply with increasing cargo loads. Long-john cargo bikes may not be suitable for narrow and crowded paths. However, the maximum allowable acceleration and deceleration are much higher compared to the other two categories of cargo bikes; therefore, these bikes can be equipped with motorized wheels that provide higher torque.

Long-tail cargo bikes are designed to carry cargo loads of around 50 kg at the rear rack. The wheelbase of the Long-tail cargo bikes is comparable to retrofitted cargo bikes and much shorter compared to Long-john cargo bikes. Therefore, these bikes do not require a greater turning radius and the self-stability and deceleration performance are similar to retrofitted cargo bikes. Moreover, the maximum acceleration values are much higher than the retrofitted cargo bikes, making it possible to use powerful hub motors to carry heavy loads. The performance metrics for Long-tail cargo bikes are less sensitive to increasing cargo loads. In contrast, Long-tail bikes have been found to require higher handlebar torque during turns. However, if the front steering geometry is carefully tuned, Long-tail cargo bikes will have much superior overall performance than retrofitted and Long-john cargo bikes for a range of light to mid-weight cargo loads.

The authors would also like to acknowledge the limitations of this research. The first limitation of the research is that the analysis is mainly based on theorical framework with cargo loads modeled as rigid blocks with simple mass and inertia properties. The second limitation is that the riders' active control input and its effect on dynamics has not been considered. Additionally, the analysis only includes two-wheeled single-track designs. This analysis could benefit from expansion to more sophisticated vehicle models that incorporate tire and frame stiffness, as well as the rider's control inputs. Further experimental work and riding trials would also be valuable in extensively exploring the rideability of cargo bikes, including those with various wheel configurations.

## Data availability statement

No data was used for the research described in the article.

## CRediT authorship contribution statement

**Milan Paudel:** Writing – review & editing, Writing – original draft, Validation, Methodology, Formal analysis, Conceptualization. **Fook Fah Yap:** Writing – review & editing, Supervision, Project administration, Methodology, Funding acquisition, Conceptualization.

## Declaration of competing interest

The authors have no conflict of interest to declare.
